# Mutation pressure mediates a pattern of substitution rates with latitude and climate in carnivores

**DOI:** 10.1002/ece3.70159

**Published:** 2024-08-27

**Authors:** Chao Zhao, Guangshuai Liu, Xiufeng Yang, Xibao Wang, Shengyang Zhou, Zhao Liu, Kangning Liu, Honghai Zhang

**Affiliations:** ^1^ College of Life Science Qufu Normal University Qufu China

**Keywords:** climate factor, latitude, metabolic rate, mutation pressure, substitution rate

## Abstract

The evolutionary patterns of the mitochondrial genome are influenced by both adaptive and nonadaptive forces, with their contributions varying among taxa. There appears to be a correlation linking mutagenesis and latitude, which could be due to differences in metabolic rates. These discrepancies in metabolic rates exhibit a positive connection with mutation pressure. On this basis, we hypothesise that nonadaptive forces play a role in the differences in mutation rates observed along latitudinal gradients. In this study, we selected widely distributed carnivores as representatives of mammals to test our hypothesis. We examined the correlations between the d*N*/d*S* ratio (ω), as well as the substitution rates (d*S* and d*N*), of 13 PCGs in the mtDNA of 122 carnivores, and the latitude and climatic factors. We found that taxa distributed in higher latitudes tend to have higher substitution rates, but not ω values indicating selective pressure. Notably, d*N* shows a strong positive correlation with d*S*, although d*S* is primarily influenced by mutation pressure, while d*N* is also influenced by effective population size (*N*_*e*_). Phylogenetic generalised least squares (PGLS) regression analyses showed that both substitution rates were correlated with climatic factors representing the temperature, precipitation and variability of climate. Based on our findings, we propose that the mutations are primarily influenced by nonadaptive forces (mutation pressure). This forms the fundamental premise for natural selection and speciation. Moreover, the correlation between substitution rates and latitudinal distribution and climate, which are outcomes of nonadaptive factors, can aid in comprehending the global distribution of species diversity.

## Graphical abstract

This study revealed a robust correlation between substitution rates and latitudinal gradients, rather than adaptation‐related parameters. And the results suggest that nonadaptive forces (mutation pressure) have a more widespread impact on mitochondrial genes compared to adaptive forces.

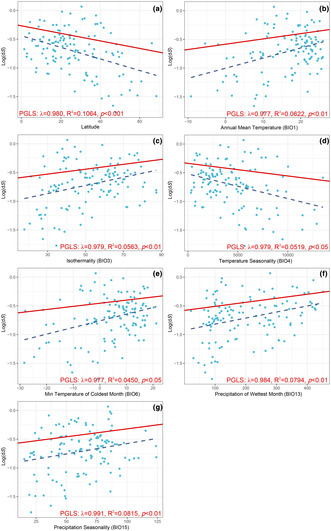

## 1 INTRODUCTION

Mitochondria are crucial organelles in eukaryotic cells, which provide up to 95% of the energy for the cells by means of oxidative phosphorylation (OXPHOS) (Das, 2006). The mitochondrial genome (mtDNA) is a small, closed, circular DNA molecule located within the mitochondrion and which generally includes 13 genes (PCGs) that encode essential proteins of the OXPHOS system (da Fonseca et al., 2008; Saraste, 1999). Because of its maternal inheritance and suitability for phylogenetic constructions and inferences about population history, mtDNA has commonly been used as a neutral evolutionary marker (Agnarsson et al., 2010; Moritz et al., 1987; Thalmann et al., 2013). However, given that the OXPHOS system has two primary physiological functions: adenosine triphosphate (ATP) production and heat generation, the assumption of selective neutrality has been challenged by some evidence suggesting that a certain amount of genetic variation within the PCGs is sensitive to natural selection (da Fonseca et al., 2008; Dowling et al., 2008; Sun et al., 2011).

Indeed, adaptive pressures play a crucial role in mtDNA evolution, and the PCGs are thought to be under strong purifying selection to reduce the fixation rate of deleterious substitutions that could affect organism fitness by directly affecting metabolic performance (Castellana et al., 2011; Palozzi et al., 2018). For example, locomotory capacity (high energy consumption) is correlated with purifying selection of mtDNA, and animals with a strong locomotor capability such as birds and mammals have undergone stronger purifying selection than those with a weak locomotor capacity (Shen et al., 2009). This relationship has been confirmed in fish and molluscs (Smith, 2016; Sun et al., 2011; Sun et al., 2018). In addition, climate has a profound effect on the evolution of mtDNA. Mitochondria can generate heat, which is advantageous for organisms living in cold climates. Therefore, mtDNA may be a strong target for climate‐driven selection. A hypothesis termed as “mitochondrial climatic adaptation” proposes that the standing mtDNA genetic variation has been shaped by natural selection imposed by the prevailing thermal climate (Camus et al., 2017). This hypothesis has been supported by some research showing that humans and other metazoans from different populations have positive selection or variation in mtDNA sequence along gradients associated with particular climates (Awadi et al., 2021; Balloux et al., 2009; Morales et al., 2015; Ruiz‐Pesini et al., 2004; Silva et al., 2014). Several lines of experimental evidence have further strengthened the hypothesis that natural selection has shaped the latitudinal distribution of mitogenomes and contributed to evolutionary adaptation under climatic stress (Camus et al., 2017; Lajbner et al., 2018).

However, the theory that nonadaptive forces, including random genetic drift and mutation pressure, are the main drivers of mtDNA evolution has been put forward to explain the variation (Jakovlić et al., 2021; Lynch, 2007; Lynch et al., 2006). Mutation pressure may be defined as the excessive vulnerability of genes, which may be caused by a variety of factors, and it has been found to result in substantial amounts of both synonymous and nonsynonymous variation among species (Jia & Higgs, 2008). There exists a positive correlation between mutation pressure and metabolic rate (Jakovlić et al., 2021). This is because the mitochondria are the main source of cellular reactive oxygen species (ROS), which are a by‐product of normal metabolism. Biochemical studies have demonstrated that species with higher metabolic rates exhibit increased rates of oxidative DNA damage and mutations (Brand, 2000; Lanfear et al., 2007; Rand, 1994). Additionally, a heightened metabolic rate results in an increased mtDNA turnover, leading to a single‐stranded state that promotes mutagenesis (Rand, 1994). In this way, the mutation of mtDNA can be exposed to both mechanisms of the effect of the metabolic rate. Rohde (1992) predicted that mutagenesis occurs more frequently as metabolic rates increase towards the equator, and this prediction could support a role for nonadaptive forces in molecular evolution. The relationship between latitudinal gradients or temperature and evolution has been demonstrated in plants, foraminifera and mammals (Allen et al., 2006; Gillman et al., 2009; Wright et al., 2006). Further, there is a growing body of evidence showing that diversification is positively correlated with the evolution of genes with a metabolic function (Lanfear et al., 2010). Since more rapid mutagenesis could increase both molecular evolution and speciation, the influence of nonadaptive forces on evolution may, to some extent, contribute to our understanding of global patterns of biodiversity.

The order Carnivora currently comprises 286 living species, distributed globally across all continents (Valkenburgh & Wayne, 2010). They span a broad spectrum of ecological niches and exhibit remarkable diversity in traits such as dietary preferences, body size and habitat choice (Huang et al., 2021; Munoz‐Garcia & Williams, 2005; Valkenburgh & Wayne, 2010). The extensive variety of carnivores has made them a popular group for evolutionary research. As carnivores are present across different latitudes and geographic locations, from equatorial deserts and forests to temperate mountain ranges and polar environments, they offer a distinct model for evaluating the effect of climate change on the mtDNA of endotherms.

Until now, prior research has chiefly concentrated on the climatic adaptation mechanisms of organisms. For example, the mtDNA of fishes living in cold climates have significantly smaller non‐substitution rates (d*N*)/substitution rates (d*S*) than tropical fishes (Sun et al., 2011). The vertebrates living in high‐altitude environment also have significantly higher d*N*/d*S* than those in low‐altitude environment (Wang et al., 2021). However, few studies have endeavoured to examine the repercussions of climate as an indirect nonadaptive force on mtDNA, specifically precipitation and climate change‐related factors (Jakovlić et al., 2021). In the present study, we initially calculated the ratio of nonsynonymous to synonymous substitution rates (ω = d*N*/d*S*), d*N* and d*S* of 13 mtDNA PCGs in 122 carnivores. We then employed analysis of variance, multiple comparisons and phylogenetic generalised least squares (PGLS) methods to ascertain the relationships between substitution rates and ω with climatic variables. Our observations suggest correlations between the substitution rates of mtDNA and climatic factors, and we provide some novel insights into the evolutionary patterns of nonadaptive processes.

## 2 MATERIALS AND METHODS

### 2.1 Species sample and climate dataset

122 available complete mitogenomes of carnivores covering 13 families and one outgroup cow (*Bos taurus*) were obtained from the NCBI GenBank database (http://www.ncbi.nih.gov, File S1). Aquatic and semi‐aquatic carnivores, such as pinnipeds and semi‐aquatic mustelids, were not included in the analyses because aquatic environments exhibit temperature differences from terrestrial environments, and water has a higher thermal conductivity. These variations may necessitate higher energy requirements for the animals to maintain their body temperature (Wei et al., 2020). The present latitude and longitude distribution data for the 122 carnivores have been sourced from the Global Biodiversity Information Facility (GBIF, 2022), with duplicate and inaccurate records were removed. The dataset consisted of species names, family names, geographical distribution information and dynamic properties for each of the species. After excluding the top and bottom 5% of latitude values for each species, the remaining values were averaged in absolute terms to obtain the final value for the following analyses. We then divided the 122 species into six latitudinal categories according to the obtained values: Group 1 (latitude <10), Group 2 (10≤ latitude <20), Group 3 (20≤ latitude <30), Group 4 (30≤ latitude <40), Group 5 (40≤ latitude <50) and Group 6 (latitude ≥50) (File S1). To investigate the relationship between evolution of mtDNA and climatic factors, the climate data at 2.5 arc‐min resolution were retrieved from the WorldClim database (http://www.worldclim.org), and the final 19 environmental variables for each species were obtained by using the capping method and then averaging them. Pearson correlation analysis was performed on all environmental variables and then ten environmental variables with low correlation (Pearson *r* < .8) were selected for further analysis: Annual Mean Temperature (BIO1), Mean Diurnal Range (BIO2), Isothermality (BIO3), Temperature Seasonality (BIO4), Max Temperature of Warmest Month (BIO5), Min Temperature of Coldest Month (BIO6), Annual Precipitation (BIO12), Precipitation of Wettest Month (BIO13), Precipitation of Driest Month (BIO14) and Precipitation Seasonality (BIO15). The carnivores were classified into four groups, from low to high, according to the environmental variables selected above. The grouping information is presented in the File S1.

### 2.2 Phylogenetic analysis and substitution rates calculation

The nucleotide sequences of the 13 mitochondrial protein‐coding genes (PCGs) were extracted using PhyloSuite (Zhang et al., 2020) and aligned in batches by MAFFT (Katoh & Standley, 2013). Then, the sequences were concatenated without stop codons for phylogenetic analysis and calculation of substitution rates. The optimal model was selected with the model finder function of PhyloSuite, and Bayesian inference (BI) approaches were employed to reconstruct the phylogenetic relationships (Ronquist et al., 2012; Zhang et al., 2020). The Bayesian posterior probabilities were estimated using the Markov Chain Monte Carlo (MCMC) method with four independent chains for 1,000,000 generations and sampling one tree every 1000 generations. The Interactive Tree of Life (ITOL) website was used to visualise the derived BI tree (Letunic & Bork, 2019). To disentangle the impacts of climate on adaptation and mutation of mitochondrial PCGs, we calculated estimates of nonsynonymous substitution rates (d*N*), synonymous substitution rates (d*S*) and the ratio of d*N* to d*S* (ω = d*N*/d*S*) for the concatenated 13 PCGs using a free ratio model implemented in PAML based on the aforementioned BI tree topology (Yang, 2007), all values were log_10_‐transformed to improve normality for statistical comparison and regression analysis.

### 2.3 Phylogenetic comparative analysis

We conducted standard statistical analyses and regression analyses to assess the relationship between different climatic factors and the substitution rates and ω values of 13 mtDNA PCGs. For standard statistical analyses, we used one‐way analysis of variance (ANOVA) and multiple comparison analysis (*t*‐test) to compare the d*N*, d*S* and ω values for different groups of carnivores according to the above grouping of latitude and climatic factors. We also investigated the potential association between latitude and climate with d*N*, d*S* and ω values using phylogenetic generalised least squares (PGLS) regression via the *Caper* package implemented in R (Orme et al., 2018). We estimated the phylogenetic signal *λ* using the maximum likelihood (ML) method. The *λ* value ranges from 0 to 1, with values close to 0 indicating phylogenetic independence and values near to 1 indicating high phylogenetic dependence. For the investigation, we obtained a time‐scaled phylogenetic tree containing the 122 carnivore species under investigation as an input tree file (Upham et al., 2019).

Owing to the necessity of maintaining the function of mitochondrial proteins, the accumulation of nonsynonymous substitutions in mtDNA PCGs is considerably lower compared to that of synonymous substitutions. Synonymous substitutions are expected to be predominantly influenced by mutation pressure, whereas nonsynonymous substitutions are expected to be influenced by mutation pressure and effective population size (*N*_*e*_) (Castellana et al., 2011; Lanfear et al., 2010). It has been shown that there is a positive correlation between synonymous and nonsynonymous variability in the genomes of several organisms, and this phenomenon supports the accuracy hypothesis that genes tend to use the most efficient codons in the process of tRNA anticodon recognition (Drummond & Wilke, 2008). To investigate whether there was also a positive correlation between d*N* and d*S* for PCGs, we performed the same association analysis using PGLS regression.

## 3 RESULTS

### 3.1 Species and climate dataset

In this research, we used a dataset comprising 122 carnivore mtDNA PCGs to perform the analyses, excluding aquatic and semi‐aquatic carnivores from the dataset, which may interfere with the climate shaping effect on mtDNA. The species and mitochondrial genome accession numbers were listed in File S1. We collected the current distribution and climate information for 122 carnivores, and these data were listed in File S1. Linear regression analysis was then performed to examine the relationship between latitude and climatic factors. In general, all ten climatic factors except Precipitation of Driest Month (BIO14) were significantly correlated with latitude values, with Temperature Seasonality (BIO4) being significantly negatively correlated with latitude values and all other climatic factors being significantly positively correlated (File S2: Table S1).

### 3.2 Phylogenetic analysis and substitution rates calculation

We reconstructed a BI phylogenetic tree of carnivores with high posterior probabilities based on 13 mitochondrial PCGs from 122 carnivores (File S2: Figure S1). In this tree, the Carnivora split into two major evolutionary lineages. The suborder Caniformia contains the six terrestrial families: Canidae, Ursidae, Ailuridae, Mephitidae, Mustelidae and Procyonidae, while the suborder Feliformia comprises seven families: Felidae, Prionodontidae, Herpestidae, Eupleridae, Hyaenidae, Viverridae and Nandiniidae. The topology of this tree was very similar to previous studies based on multiple nuclear genes and mitogenomes (Agnarsson et al., 2010; Eizirik et al., 2010; Hassanin et al., 2021).

The nonsynonymous substitution rates (d*N*), synonymous substitution rates (d*S*) and ω values of the concatenated 13 PCGs for 122 terminal branches were evaluated by a free ratio model depending on the topology of the BI tree constructed above (File S1). The results showed that the d*S* values ranged from 0.01689 to 1.1678 and the d*N* values ranged from 0.00119 to 0.04316, with the d*N* values being significantly lower than the d*S* values (*p* < .001). The ω values were less than 0.1142, indicating that the PCGs of carnivores were under strong purifying selection during evolution.

### 3.3 Statistical comparisons of substitution rates of different groups

**FIGURE 1:**
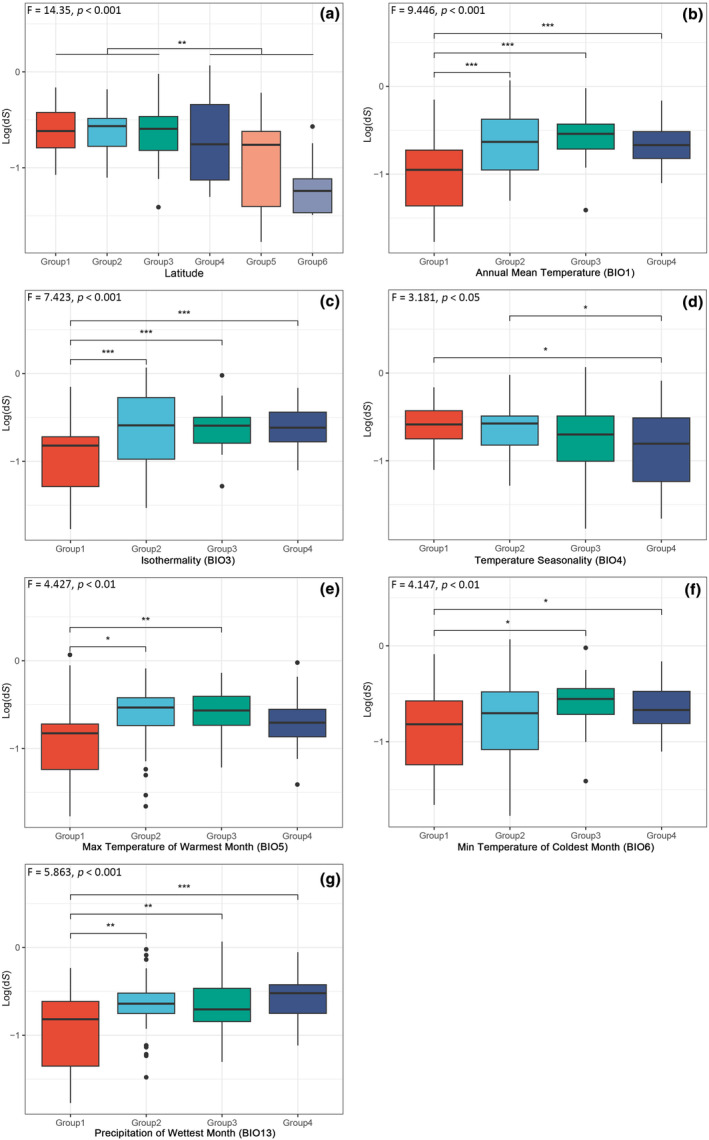
Statistical comparisons of synonymous substitution rates (d*S*) grouped by latitude (a) and climatic factors (b–g). X axis: Categories of latitude and climatic factors. Y axis: DS of 13 mtDNA PCGs after log transformation. Species were classified into six latitudinal categories based on latitude values: Group 1 (latitude <10), Group 2 (10≤ latitude <20), Group 3 (20≤ latitude <30), Group 4 (30≤ latitude <40), Group 5 (40≤ latitude <50) and Group 6 (latitude ≥50). Species were classified into four climatic categories according to the rankings of climatic factors. The *p*‐values located in the top left corner of the chart were extracted from results of ANOVA analyses. Pairwise comparisons were performed through *t*‐test method, *, **, and *** above horizontal lines represent statistically significant (*p* < .05, *p* < .01, *p* < .001).

To test the relationship between climate and substitution rates and ω values of PCGs, we employed ANOVA to compare the d*S*, d*N* and ω from different groups of latitudinal categories and climatic factors. In general, the comparative analyses of d*N* and d*S* values showed similar results. The d*S* and d*N* values were significantly different among the 122 carnivores grouped into six latitudinal categories (d*S*, *F* = 5.704, *p* < .001; d*N*, *F* = 5.408, *p* < .001), with Group 6 species having the lowest mean d*S* and d*N* values, followed by Group 5 and Group 4. The *t*‐test results showed that substitution rates were significantly lower in the latitude >30 category than in the latitude <30 category (*p* < .001, Figure 1a, File S2: Figure S2a). In the grouping of environmental factors, the ANOVA analyses showed that the d*S* and d*N* values were significantly different among the groups based on Annual Mean Temperature (BIO1), Isothermality (BIO3), Temperature Seasonality (BIO4), Max Temperature of Warmest Month (BIO5), Min Temperature of Coldest Month (BIO6) and Precipitation of Wettest Month (BIO13) (Figure 1, File S2: Figure S2). Group 1 had significantly lower d*S* and d*N* values than other groups in Annual Mean Temperature (BIO1), Isothermality (BIO3) and Precipitation of Wettest Month (BIO13) (*p* < .05), whereas in Max Temperature of Warmest Month (BIO5) the substitution rates of Group 1 were only significantly lower than those of Group 2 and Group 3 (*p* < .05). In Temperature Seasonality (BIO4), Group 4 had significantly lower d*S* values than Group 1 and Group 2 (*p* < .05), and Group 3 also had significantly lower d*N* values than Group 1 and Group 2. In addition, the ANOVA analysis showed that the substitution rates of Group 1 were significantly lower than those of Group 3 and Group 4 in Min Temperature of Coldest Month (BIO6) (*p* < .01, Figure 1f, File S2: Figure S2f). The ω values only displayed significant differences across latitudinal categories in the ANOVA analysis (*F* = 2.509, *p* < .05). However, no pairwise significant difference was identified in the comparison. No significant differences were found among the categories of each climatic factor.

### 3.4 Correlation between latitude and climatic factors and substitutions rates

**FIGURE 2:**
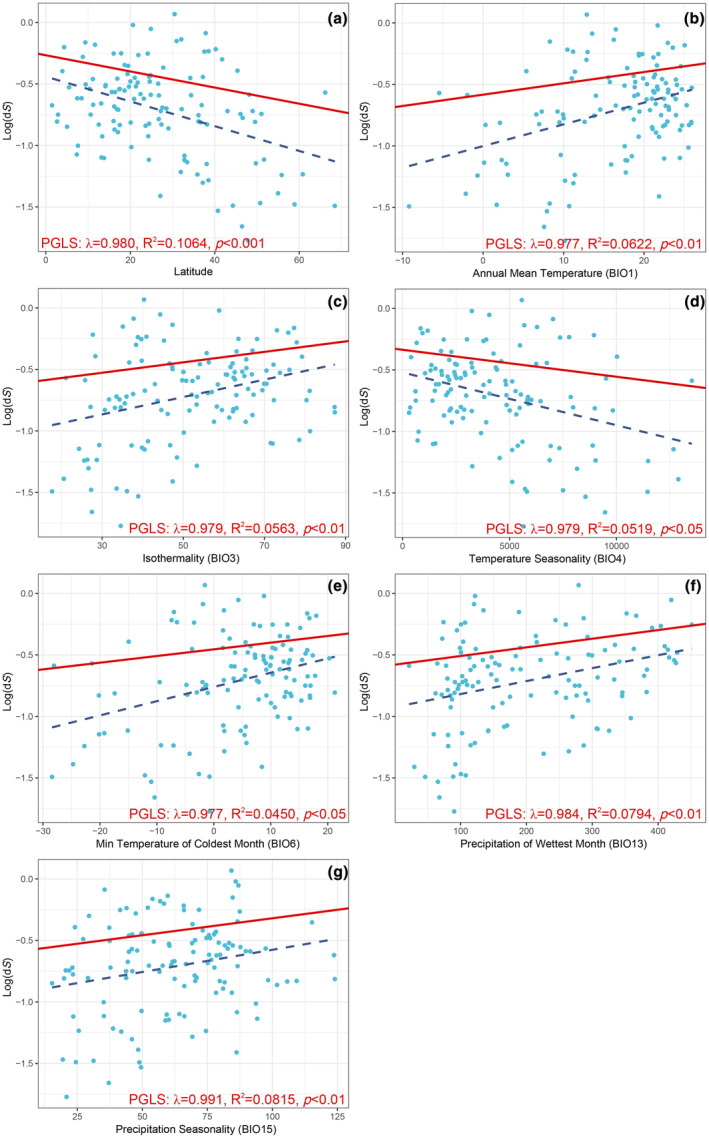
Regression analyses between d*S* and latitude (a) and climatic factors (b–g) by PGLS. The solid lines represent the regressions from the PGLS methods, and the dashed lines represents regression lines fitted to the data (*p* < .05), with the slope and intercept values displayed.

We first carried out a PGLS regression analysis on the relationship between the d*N* and d*S* values. According to the result, the d*S* values showed an extremely significant correlation with the d*N* values (*R* ^2^ = 0.8081, *p* < .001) (File S2: Figure S3). We further investigated the potential relationship between latitude values and climatic factors of each carnivore and substitution rates of PCGs. The findings indicate that the d*S* (*R* ^2^ = 0.1064, *p* < .001) and d*N* (*R* ^2^ = 0.1745, *p* < .001) values display a significantly negative correlation with latitude values (Figure 2a, Table 1, File S2: Figure S4a). The observation indicates that carnivores distributed at lower latitudes tend to have higher d*S* and d*N* values. For the climatic factors, PGLS regression analyses revealed that both of the d*S* and d*N* values were significantly associated with Annual Mean Temperature (BIO1), Temperature Seasonality (BIO4), Min Temperature of Coldest Month (BIO6), Precipitation of Wettest Month (BIO13) and Precipitation Seasonality (BIO15) (Figure 2, Table 1, File S2: Figure S4). Also, the d*S* values were significantly associated with Isothermality (BIO3), and the d*N* values were significantly associated with Mean Diurnal Range (BIO2), Max Temperature of Warmest Month (BIO5) and Annual Precipitation (BIO12) (Table 1, File S2: Figure S4). According to the correlation slope, most of the substitution rates of PCGs showed a positive correlation with the climatic factors, except for the values of both substitution rates which displayed a negative correlation with Temperature Seasonality (BIO4) (File S2: Figure S4d). In the PGLS regression analyses, exploring the correlation between ω values and latitude values and climatic factors, however, no significant correlations were identified between climatic factors and ω. In summary, the PGLS analyses indicated that substitution rates were significantly correlated with latitude values and may be influenced by some climatic factors.

**TABLE 1:** Significant results of PGLS analyses for substitutions rates with latitude and climatic factors.

Response variable	Predictor variable	Slope	*λ*	*R* ^2^	*p*
d*S*	Latitude	−0.00656	0.980	.1064	<.001***
d*N*		−0.00852	0.899	.1745	<.001***
d*S*	Annual Mean Temperature (BIO1)	0.00921	0.977	.0622	<.01**
d*N*		0.01194	0.871	.0945	<.001***
d*S*	Mean Diurnal Range (BIO2)	0.02016	0.984	.0280	.065
d*N*		0.02305	0.921	.0326	<.05*
d*S*	Isothermality (BIO3)	0.00425	0.979	.0563	<.01**
d*S*	Temperature Seasonality (BIO4)	−0.00002	0.979	.0519	<.05*
d*N*		−0.00003	0.887	.0954	<.001***
d*S*	Max Temperature of Warmest Month (BIO5)	0.00864	0.980	.0303	.055
d*N*		0.01005	0.899	.0354	<.05*
d*S*	Min Temperature of Coldest Month (BIO6)	0.00543	0.977	.0450	<.05*
d*N*		0.00751	0.878	.0774	<.01**
d*S*	Annual Precipitation (BIO12)	0.00008	0.978	.0287	.062
d*N*		0.00010	0.902	.0507	<.05*
d*S*	Precipitation of Wettest Month (BIO13)	0.00070	0.984	.0794	<.01**
d*N*	0.00074	0.908	.0830	<.01**
d*S*	Precipitation Seasonality (BIO15)	0.00276	0.991	.0815	<.01**
d*N*	0.00250	0.925	.0518	<.05*

*p < .05; **p < .01; ***p < .001.

## 4 DISCUSSION

In this research, we focused on the association between the latitudinal gradient of 122 carnivores and the substitution rates and ω values of 13 PCGs in mtDNA. We also investigated the correlation between the climatic factors that closely related to latitude and the substitution rates and ω values. We demonstrated that the substitution rates of 13 PCGs from different carnivores were significantly associated with latitudinal distribution, and species from low latitudes had relatively higher substitution rates than those from high latitudes, which is consistent with the central idea of Rohde's prediction (Rohde, 1992). Our results suggest that climatic factors associated with animal metabolic rate may be a type of nonadaptive force influencing substitution rates. In the subsequent section, we elaborate on these findings in greater detail.

### 4.1 Nonadaptive forces are primary drivers of mitochondrial genome evolution

Numerous studies have demonstrated that mitochondrial genes are characterised by high mutational pressure (Jakovlić et al., 2021; Lynch et al., 2006). Nonsynonymous substitutions of PCGs were found to evolve at a slower pace than synonymous substitutions (Castellana et al., 2011). Synonymous substitutions tend to have very small selective effects and are usually considered to be neutral mutations. Therefore synonymous substitution rates, which were used to estimate the number of substitutions without changing the amino acid sequence, are predominantly determined by the mutation pressure (Lanfear et al., 2010). The nonsynonymous substitution rates estimate the number of substitutions that change the amino acid sequence and turn out to be influenced by the mutation pressure and effective population size (*Ne*) (Lanfear et al., 2010). The ω value serves as an indicator of selection pressure and has been found to have a significant association with *Ne* (Kosiol et al., 2008). In our analyses, we found no significant correlation between ω values and either latitude or climatic factors (File S2: Table S2), so it seems plausible that the adaptive forces exerted on animals by latitude and associated climatic factors are not predominantly evolutionary drivers. Furthermore, the results show that both d*N* and d*S* values are significantly correlated with latitude and climatic factors. It is also worth noting that d*N* is highly correlated with d*S*, suggesting mutation pressure may account for the variation in d*N*. Taken together, we hypothesise that the nonadaptive force of associated mutation pressure may have become the main driver of evolution, providing a large amount of raw material for drift and natural selection.

### 4.2 Substitution rates of carnivores are linked to latitudinal gradient

Rohde's prediction was that mutagenesis would be more common near the equator than it would be at higher latitudes (Rohde, 1992). This prediction has been confirmed in subsequent studies on plants and foraminifera (Allen et al., 2006; Wright et al., 2006). The mechanistic explanations for the relationship between mutagenesis and latitudes were mainly attributed to the effect of metabolic rates. It has suggested that species living within lower latitudes have more biologically available energy and increased productivity, which increases the metabolic rates (Allen et al., 2006; Gillman & Wright, 2006; Wright et al., 2006). Besides, the mutation pressure was thought to be positively correlated with metabolic rates, as high metabolic rates may influence mutagenesis by inducing oxidative DNA damage and accelerating the DNA replication rate (Brand, 2000; Lanfear et al., 2007; Rand, 1994). It has been suggested that there is no clear correlation between microevolution of endotherms and latitudinal gradients (Mittelbach et al., 2007; Weir & Schluter, 2007). However, Gillman et al. (2009) discovered that mammals residing in lower latitudes with warmer climates exhibit higher substitution rates of the mitochondrial cytochrome *b* gene compared to species inhabiting higher latitudes, demonstrating that Rohde's prediction also applies to endotherms. The same study also found that the temperature affected by elevation was also correlated with substitution rates, and they attributed changes in substitution rates to the ambient thermal environment or indirectly controlled by the coevolving ectotherms living in the same habitats. Our results indicating that substitution rates (including d*N* and d*S*) were significantly lower in species distributed in high latitude than in low latitude (Figure 1a, File S2: Figure S2a), and that substitution rates were also significantly negatively correlated with latitude values (Figure 2a, Table 1, File S2: Figure S4a), are consistent with the above prediction. Our study weaves the variation in substitution rates, latitude and metabolic rate into a single unifying theory and provides novel molecular evidence for Rohde's prediction.

Empirical evidence has shown that the tropics have more species than temperate regions, and that more species accumulate per unit time at tropical latitudes, leading to a latitudinal gradient in species diversity (Gillman et al., 2009; Weir & Schluter, 2007; Wright et al., 2006). Available energy and productivity may be the driver of molecular evolution, and significant correlations between rates of molecular evolution (genes with metabolic functions) and diversity have been found in a range of taxa (Davies et al., 2004; Lanfear et al., 2010). The link between molecular evolution and species diversity is mediated by the mutation, for increased mutation rates could accelerate rates of speciation by generating genetic differences between diverging lineages that lead to hybrid incompatibility (Orr & Turelli, 2001; Pagel et al., 2006). The synonymous substitution rate is predominantly determined by the mutation rate. Besides, our analyses showed that the d*N* values are significantly correlated with the d*S* values, and some studies have also reported that an increased mutation rate could increase the d*N* (Castellana et al., 2011; Yang & Nielsen, 1998), so we can make an inference that there is an association between the substitution rate and the latitudinal gradient of species diversity. In this case, we could speculate that diversity of carnivore is negatively correlated with latitude, which is consistent with the global latitudinal distribution pattern of mammals (Kaufman & Willig, 1998; Willig & Presley, 2018). Our analyses of substitution rates in relation to the latitudinal gradient allow us to make some inferences about the possible molecular mechanism of the latitudinal gradient in species diversity and provide new insights into understanding global patterns of species diversity.

### 4.3 Substitution rates of carnivores are associated with climatic factors

Many climatic factors change with latitude, suggesting that climate has been important in the evolution of mtDNA (Lovegrove, 2003). We further analysed the relationship between a range of climatic factors (including temperature and precipitation) and substitution rates. Both temperature and water availability are important climatic determinants of productivity, which is positively correlated with metabolic rates in mammals (Gillman & Wright, 2006; Wright et al., 2006). For precipitation‐related climatic factors, our results show that Precipitation of Wettest Month (BIO13) is positively correlated with substitution rates. Furthermore, the group with the least amount of precipitation has significantly lower substitution rates than the other groups. Mammals in areas with low precipitation (e.g., deserts) have lower metabolic rates compared to animals in mesic areas, mainly because primary productivity and food availability are directly associated with rainfalls (Lovegrove, 2000; White et al., 2007). We hypothesise that a reduction in precipitation may indirectly influence the substitution rates of carnivores in less rainy environments, potentially through an effect on metabolic rates.

For temperature‐related climatic factors, Annual Mean Temperature (BIO1) was found to be positively correlated with substitution rates. The group with the lowest temperature, Group 1, exhibited considerably lower substitution rates than the remaining groups. Correlation analysis showed a strong correlation between annual mean temperature and latitude (*r* = −0.84, *p* < .001). It implies that the association between mutation rates and annual mean temperature may arise due to reduced metabolic rate, as productivity falls at higher latitudes (Gillman et al., 2009; Wright et al., 2006). In addition, animals are expected to increase the efficiency of endogenous heat production to improve cold tolerance in relatively cold environments. Mitochondria produce ATP, heat and ROS through the oxidative phosphorylation system, and it has been shown that the balance between ATP, heat and ROS production depends on proton leakage, which tends to produce more heat and less ROS in cold environments (Lowell & Spiegelman, 2000; Murphy, 2009; Sunnucks et al., 2017). This not only facilitates the enhancement of cold tolerance but also explains why the substitution rates in terms of ROS production are lower (Lamb et al., 2018; Stier et al., 2014). Surprisingly, despite the positive correlation between temperature and substitution rates, we found that the Group 4 of Annual Mean Temperature (BIO1), Max Temperature of Warmest Month (BIO5) and Min Temperature of Coldest Month (BIO6) had slightly lower substitution rates compared to those in Group 3. Animals tend to decrease their metabolism at extreme temperatures by reducing their activity and endogenous heat production (Lovegrove, 2000, 2003; White et al., 2007). This can potentially impact the substitution rates.

Furthermore, we detected a positive correlation between Isothermality (BIO3) and substitution rates. Isothermality represents thermal stability with respect to annual temperature variations. Isothermality indirectly affects the distribution of wetland habitat by influencing seasonal hydrological cycles, and decreasing isothermality may generate a more variable hydrograph (Garris et al., 2015). We found that isothermality exhibited the most significant correlation with latitude of all climatic factors studied. This suggests that isothermality may influence habitat type to some extent along latitudinal gradients, leading to latitudinal trends in energy available to animals. This, in turn, is positively correlated with substitution rates. For variability‐related climatic factors, we also found that the substitution rates were positively correlated with the values of Mean Diurnal Range (BIO2) and Precipitation Seasonality (BIO15). Animal metabolic rates exhibit plasticity under different environmental conditions (McKechnie, 2008). For example, metabolic rates increase following an acute rise in temperature (Seebacher et al., 2015). Metabolic plasticity may increase resistance to climate change. The drastic changes in climate may contribute to major energetic challenges for animals, resulting in reversible changes in metabolic rate and increases in the efficiency of ATP production (Norin & Metcalfe, 2019). In this case, this more coupled system with less proton leakage is associated with the production of increased ROS, which may lead to more mutations.

## 5 CONCLUSIONS

Adaptive and nonadaptive evolutionary forces contribute variably to the evolution of mitochondria among different lineages, which has led to inconsistent and contradictory results in mitogenomic evolutionary research. The results of this study reveal a robust positive correlation between synonymous and non‐synonymous substitution rates, as well as a correlation between substitution rates and latitudinal gradients, rather than adaptation‐related parameters. These results suggest that nonadaptive forces (mutation pressure) have a more widespread impact on mitochondrial genes compared to adaptive forces. The findings reinforce the theory that nonadaptive forces are the main drivers influencing the evolution of mitochondrial genes. Metabolic rate, which is positively correlated with mutation pressure, may indirectly explain the relationship between substitution rates and latitudinal gradients and climatic factors. This observation upholds Rohde's prediction that mutagenesis becomes more common as metabolic rates rise closer to the equator. Our study reveals the evolutionary pattern of mitochondrial genes in mammals, represented by carnivores, and provides new evidence for understanding the global distribution of species diversity.

## AUTHOR CONTRIBUTIONS

**Chao Zhao:** Conceptualization (equal); data curation (equal); methodology (equal); project administration (equal); visualization (equal); writing – original draft (equal). **Guangshuai Liu:** Conceptualization (equal); methodology (equal); project administration (equal); validation (equal); writing – original draft (equal). **Xiufeng Yang:** Methodology (equal); project administration (equal); validation (equal); writing – original draft (equal). **Xibao Wang:** Formal analysis (equal); resources (equal); software (equal). **Shengyang Zhou:** Formal analysis (equal); software (equal). **Zhao Liu:** Data curation (equal); formal analysis (equal); resources (equal). **Kangning Liu:** Formal analysis (equal); investigation (equal); resources (equal). **Honghai Zhang:** Conceptualization (equal); funding acquisition (equal); methodology (equal).

## CONFLICT OF INTEREST STATEMENT

The authors declare no competing interests.

## Supporting information


File S1.

File S2.

## Data Availability

123 available complete mitochondrial genomes of carnivores and *Bos taurus* were obtained from the NCBI GenBank database (http://www.ncbi.nih.gov/). The species and mitochondrial genome accession numbers were listed in File S1. The present distribution data for the 122 carnivores were sourced from the Global Biodiversity Information Facility (https://www.gbif.org/en/), and the climate data were retrieved from the WorldClim database (http://www.worldclim.org).
