# Correction to “Mutation pressure mediates a pattern of substitution rates with latitude and climate in carnivores”

**DOI:** 10.1002/ece3.72229

**Published:** 2025-09-29

Zhao, C., Liu, G., Yang, X., Wang, X., Zhou, S., Liu, Z., Liu, K., & Zhang, H. (2024). Mutation pressure mediates a pattern of substitution rates with latitude and climate in carnivores. *Ecology and Evolution*, 14, e70159. https://doi.org/10.1002/ece3.70159

In Lines 10–11 of the Abstract section, the text “We found that taxa distributed in higher latitudes tend to have higher substitution rates” was incorrect.

This should have read: “We found that taxa distributed in lower latitudes tend to have higher substitution rates”

We apologize for this error.

