# Supplementary material for: Mutation pressure mediates a pattern of substitution rates with latitude and climate in carnivores
Source: Ecol Evol. 2024 Aug 27;14(8):e70159. doi: 10.1002/ece3.70159 (PMC11347990; doi:10.1002/ece3.70159)
Supplement: Supplementary file 2 — File S2. [file ECE3-14-e70159-s001.docx]

Table S1. Correlation analyses of Latitude and Climatic Factors. Climatic factors are ordered by the *r*.

| Climatic Factors | ***r*** | ***p*-value** | **signif.** |
| --- | --- | --- | --- |
| BIO3 | -0.9219 | <0.0001 | *** |
| BIO4 | 0.8772 | <0.0001 | *** |
| BIO6 | -0.8570 | <0.0001 | *** |
| BIO1 | -0.8410 | <0.0001 | *** |
| BIO5 | -0.5733 | <0.0001 | *** |
| BIO13 | -0.5634 | <0.0001 | *** |
| BIO12 | -0.5375 | <0.0001 | *** |
| BIO15 | -0.3398 | 0.0001 | *** |
| BIO2 | -0.2019 | 0.0258 | * |
| BIO14 | -0.1529 | 0.0927 |  |

**Table S2** **Results of PGLS analyses for substitution rates and ω values with latitude and climatic factors.**

| Response variable | Predictor variable | Slope | λ | R2 | p |
| --- | --- | --- | --- | --- | --- |
| dS | Precipitation of Driest Month (BIO14) | - | - | - | - |
| dN |  | -0.00011 | 0.919 | 0.0001 | 0.906 |
| ω | Latitude | -0.00026 | 0.503 | 0.0007 | 0.773 |
| ω | Annual Mean Temperature (BIO1) | -0.00092 | 0.471 | 0.0023 | 0.598 |
| ω | Mean Diurnal Range (BIO2) | 0.00274 | 0.491 | 0.0018 | 0.646 |
| ω | Isothermality (BIO3) | 0.00090 | 0.540 | 0.0103 | 0.267 |
| ω | Temperature Seasonality (BIO4) | 0.00000 | 0.498 | 0.0006 | 0.797 |
| ω | Max Temperature of Warmest Month (BIO5) | -0.00161 | 0.475 | 0.0034 | 0.525 |
| ω | Min Temperature of Coldest Month (BIO6) | -0.00038 | 0.479 | 0.0008 | 0.758 |
| ω | Annual Precipitation (BIO12) | -0.00001 | 0.474 | 0.0021 | 0.612 |
| ω | Precipitation of Wettest Month (BIO13) | -0.00011 | 0.462 | 0.0087 | 0.306 |
| ω | Precipitation of Driest Month (BIO14) | 0.00034 | 0.497 | 0.0044 | 0.469 |
| ω | Precipitation Seasonality (BIO15) | -0.00037 | 0.489 | 0.0039 | 0.492 |


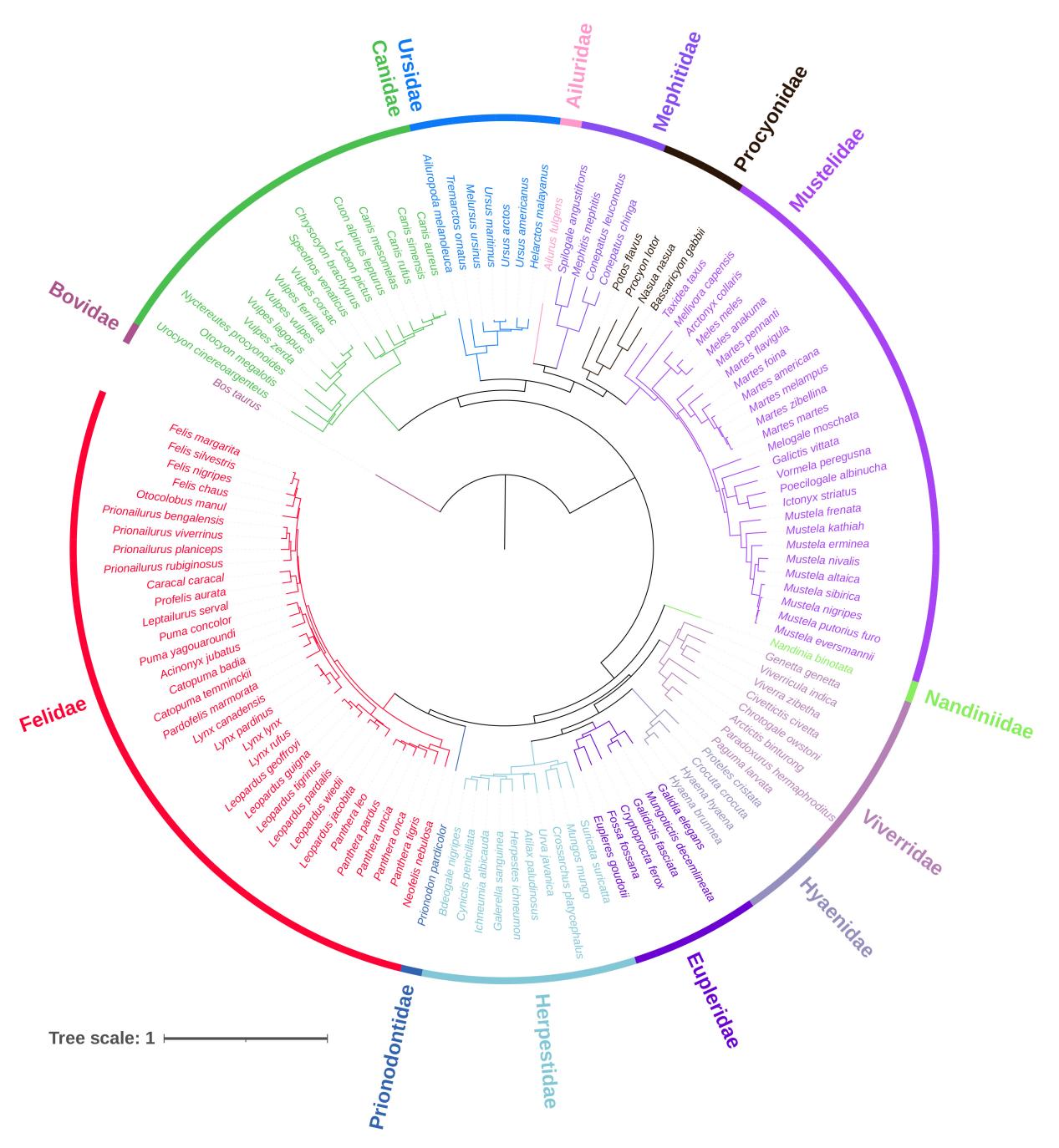


**Figure S1 The BI phylogenetic tree of 122 carnivores based on 13 PCGs of mitochondrial genomes**

**
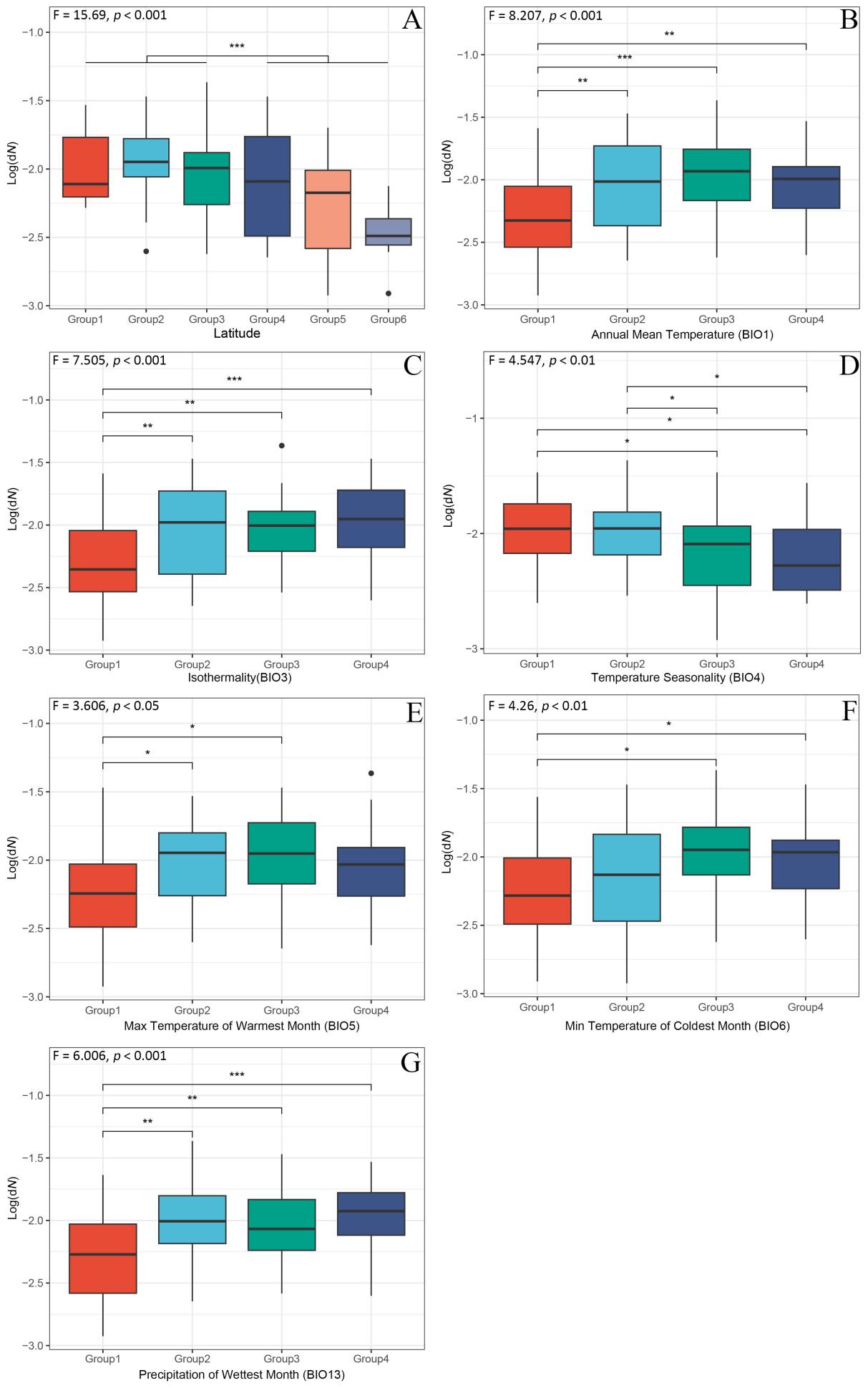
**

**Figure S2 Statistical comparisons of nonsynonymous synonymous substitution rates (d*N*) grouped by** **latitude (A) and** **climatic factors (B-G).**


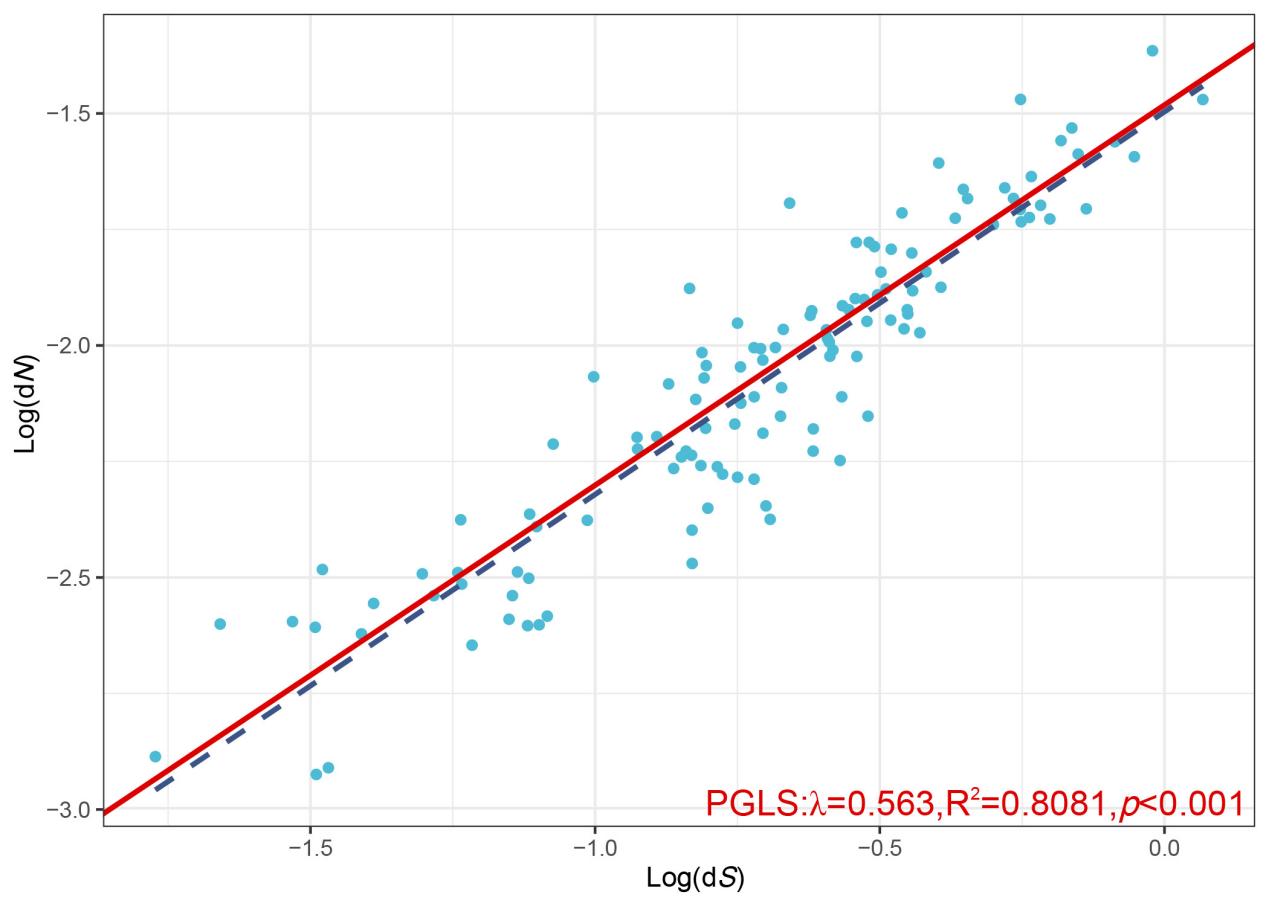


**Figure S3 Regression analyses between d*S* and d*N* by PGLS.**


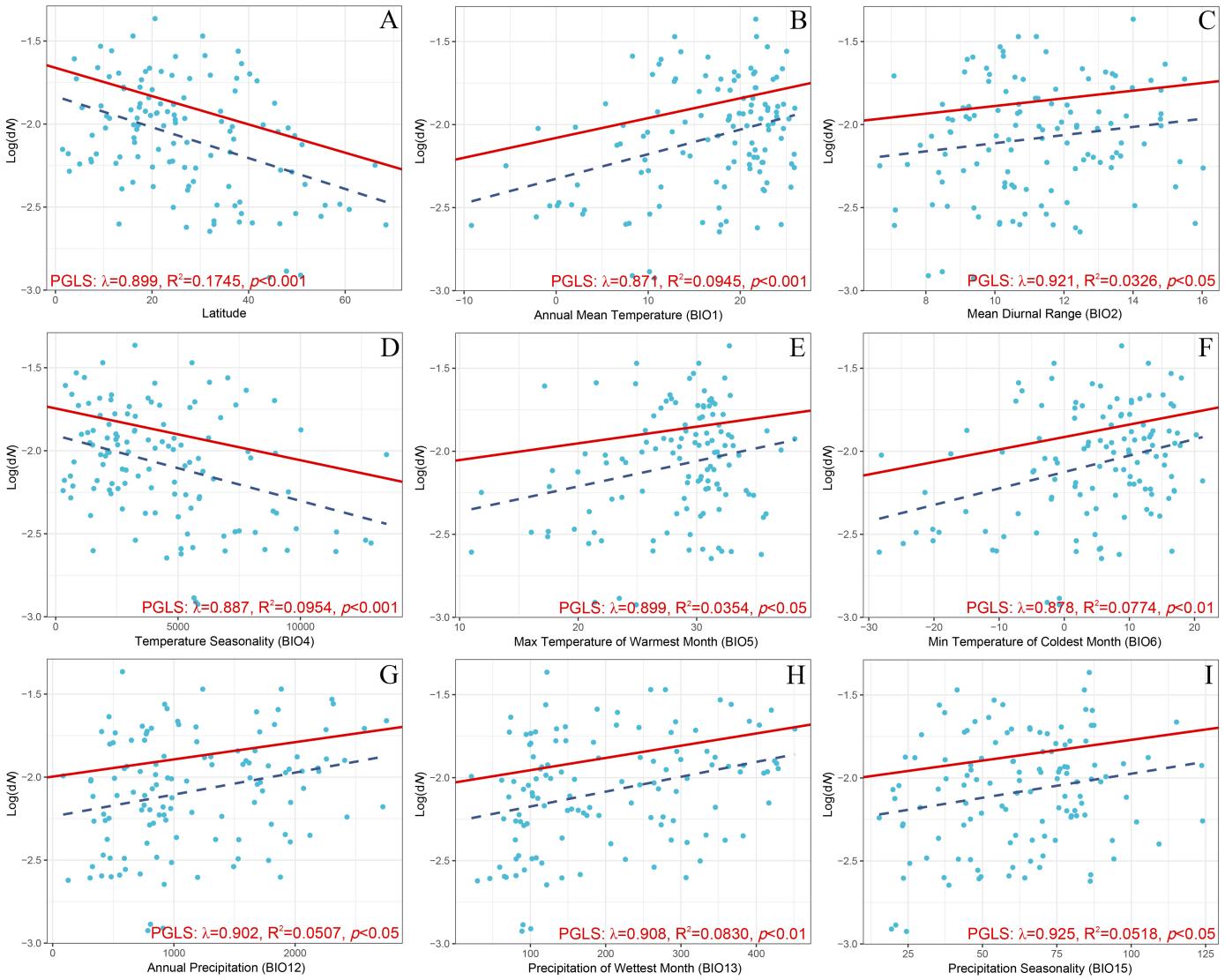


**Figure S****4** **Regression analyses between d*N* and latitude (A) and climatic factors (B-I) by PGLS.** **The solid lines represent the regressions from the PGLS methods, and the dashed lines represents regression lines fitted to the data (*p*<0.05), with the slope and intercept values displayed.**
